# Anti-nociceptive effect of *Faecalibacterium prausnitzii* in non-inflammatory IBS-like models

**DOI:** 10.1038/srep19399

**Published:** 2016-01-18

**Authors:** S. Miquel, R. Martín, A. Lashermes, M. Gillet, M. Meleine, A. Gelot, A. Eschalier, D. Ardid, L. G. Bermúdez-Humarán, H. Sokol, M. Thomas, V. Theodorou, P. Langella, F. A. Carvalho

**Affiliations:** 1 INRA, Commensal and Probiotics-Host Interactions Laboratory, UMR 1319 Micalis, F-78350 Jouy-en-Josas, France; 2 AgroParisTech, UMR1319 Micalis, F-78350 Jouy-en-Josas, France; 3 Laboratoire Microorganismes: Génome et Environnement, UMR CNRS 6023, 63000 Clermont-Ferrand, France; 4 Université d’Auvergne, 63000, Clermont-Ferrand, France; 5 INSERM 1107 Neuro-Dol, 630000 Clermont-Ferrand, France; 6 Neuro-Gastroenterology and Nutrition Team, TOXALIM, UMR 1331-INRA/INP/UPS, F-31931 Toulouse, France; 7 APHP, Hôpital Saint Antoine – Service de Gastroentérologie et nutrition, F-75012 Paris, France; 8 Sorbonne Universités, UPMC Univ Paris 06, LBM, 27 rue de Chaligny, F-75012 Paris, France; 9 INSERM-ERL 1157 and Inflammation-Immunopathology-Biotherapy Department (DHU i2B), CHU Saint-Antoine 27 rue de Chaligny, F-75012 Paris, France; 10 CNRS, UMR 7203 LBM, F-75005 Paris, France

## Abstract

Visceral pain and intestinal dysbiosis are associated with Irritable Bowel Syndrome (IBS), a common functional gastrointestinal disorder without available efficient therapies. In this study, a decrease of *Faecalibacterium prausnitzii* presence has been observed in an IBS-like rodent model induced by a neonatal maternal separation (NMS) stress. Moreover, it was investigated whether *F. prausnitzii* may have an impact on colonic sensitivity. The A2-165 reference strain, but not its supernatant, significantly decreased colonic hypersensitivity induced by either NMS in mice or partial restraint stress in rats. This effect was associated with a reinforcement of intestinal epithelial barrier. Thus, *F. prausnitzii* exhibits anti-nociceptive properties, indicating its potential to treat abdominal pain in IBS patients.

Irritable Bowel Syndrome (IBS), a gastrointestinal (GI) pathology, frequently associated with psychological distress with a worldwide prevalence of 5–20%, accordingly to the diagnosis criteria, is defined by altered bowel habits, chronic abdominal pain, absence of detectable structural colon abnormalities and increased gut permeability^1^. Colonic hypersensitivity (CHS) is considered as a major factor playing a role in IBS physiopathology and is clinically revealed by increased perception of colorectal distension^2^. This common complaint is a crucial feature because of its significant impact on IBS patients’ quality of life and lack of efficient therapies. Evidence is growing to support that low grade intestinal inflammation is involved in the pathophysiology of IBS^3^.

Abnormalities in colonic microbiota have been suspected in IBS patients with bloating, and altered intestinal motility or sensitivity^4^,^5^. Growing evidence suggest that at least subgroups of IBS patients have an altered gut microbiota composition or dysbiosis^6^,^7^. Some articles report a decrease in *Faecalibacterium prausnitzii*, a feature common to several intestinal disorders^8^,^9^. *F. prausnitzii* is an extremely oxygen sensitive commensal butyrate producer bacterium of the gut microbiota, recognized as a biomarker of intestinal health^9^. *F. prausnitzii* and its supernatant (SN) have demonstrated anti-inflammatory effects in both acute^10^,^11^ and chronic^12^ chemically-induced colitis models.

In this context, this study proposed to investigate the effects of *F. prausnitzii* in two animal models of CHS induced by chronic or acute stress: Neonatal Maternal Separation (NMS)^13^ in mice and Partial Restraint Stress (PRS)^14^ in rats. Hypersensitive animals treated with *F. prausnitzii* but not with its culture SN showed a decreased colonic sensitivity in response to a colorectal distension (CRD).

## Methods

### Bacterial strains and growth conditions

*Faecalibacterium prausnitzii* A2-165 strain (DSMZ collection, Braunschweig, Germany) (DSM N° 17677) was grown in LYBHI medium (Brain-heart infusion medium supplemented with 0.5% yeast extract) (Difco, Detroit, USA) supplemented with 1 mg/ml cellobiose (Sigma-Aldrich Chemie GmbH, Buchs, Switzerland), 1 mg/ml maltose (Sigma-Aldrich), and 0.5 mg/ml cysteine (Sigma-Aldrich) at 37 °C in an anaerobic chamber. *F. prausnitzii* inoculums preparation for gavages, contain viable bacteria, as previously described^11^. *F. prausnitzii* supernatant (SN) was recovered by centrifugation, filtered with 0.45 μm filter (VWR, Haasrode, Belgium) and stored at −80 °C until further use. When appropriated, 1.5% (w/v) agar (Difco) was added to the liquid medium.

### Animals

Concerning mice experiments, C57Bl/6 J mice (Janvier laboratories, Le Genest Saint Isle, France) were used to obtain male pups to treat as described below in University of Clermont-Ferrand animal facility. Females Wistar rats (Janvier Le Genest Saint Isle, France) weighing 200–225 g were housed individually in a temperature controlled room (21 +/−1 °C) under standard conditions in Toxalim’s installations (TOXALIM, INRA, Toulouse, France). Toxalim animal facility (INRA, UMR 1331, Toulouse, France; agreement n° B31.555.13) and University of Clermont-Ferrand animal facility (Université d’Auvergne, Clermont-Ferrand, France; agreement n°B.63 113.15) are licensed by the French Ministry of Agriculture. All experiments were performed according to the ethical guidelines set out by the International Association for the Study of Pain (IASP), complied with the European Union regulation and were approved by ethics committees: the local committees C2EA-02 of Clermont-Ferrand (approvals CE110-12 and CE111-12) and the regional Midi-Pyrénées committee C2EA-86 (approval TOXCOM/0037/VT VT).

### Animal preparation, stress induction and treatments

#### Neonatal Maternal Separation (NMS)

**Figure 1:**
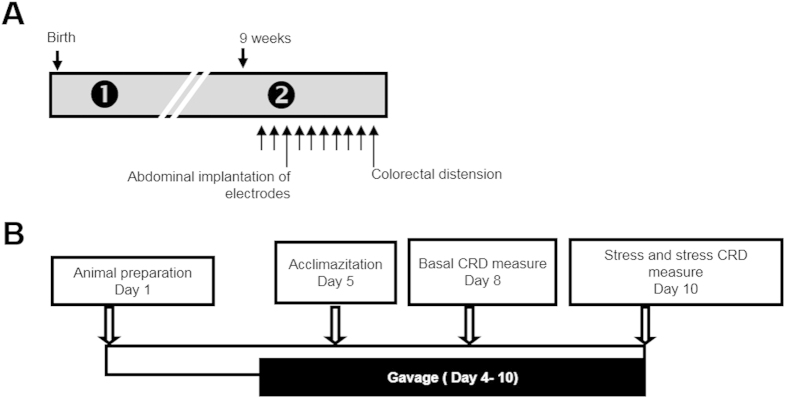
Time course protocol for NMS (A) and PRS (B) experiments.

(Fig. 1A): After birth, wild-type C57Bl/AJ pups were isolated from their mother from P2 to P14, three hours per day (from 9:00 a.m. to 12:00 p.m.). These mice was named Neonatal Maternal Separated (NMS) mice compared to Non-Handled (NH) mice. Pups were then left with their mothers up to weaning (P21). All experiments were performed on nine-week-old male mice. During ten days, 0.2 ml of ±1 × 10^10^ CFU, PBS or *F. prausnitzii* SN were daily intragastrically administrated. The study groups are as follows: control group (PBS), free-bacteria culture medium (LYBHI), *F. prausnitzii* A2-165 strain (A2-165), *F. prausnitzii* A2-165 supernatant (SN).

#### Partial restraint stress (PRS)

(Fig. 1B): Animal preparation was performed as previously described^14^. Briefly, under general anesthesia induced by *i.p*. administration of 0.6 mg/kg acepromazine (Calmivet, Vetoquinol, Lure, France) and 120 mg/kg ketamine (Imalgene 1000, Merial, Lyon, France), female Wistar rats as previously described^15^ were equipped with three groups of three NiCr wire electrodes (60 cm in length, 80 nm in diameter) implanted into the abdominal external oblique muscle, 2 cm above the inguinal ligament. Electrodes were exteriorized at the neck level by a glass tube attached to the skin. During the ten days previous to the stress, 1 ml of ±1 × 10^9^ CFU, PBS or *F. prausnitzii* SN were daily intragastrically administrated. The study groups are as follows: control group (PBS), free-bacteria culture medium (LYBHI), *F. prausnitzii* A2-165 strain (A2-165), *F. prausnitzii* A2-165 supernatant (SN). All stress sessions were performed at the same time of the day (between 10 am and 12 pm) to minimize any influence of circadian rhythms. Stresses were performed using the wrap partial restrain stress model which is a mild non-ulcerogenic stressor^16^. Animals were lightly anesthetized as previously described^17^ with ethyl-ether and their fore shoulders, upper forelimbs and thoracic trunk were draped in a confining harness of paper tape to restrict, but not to prevent, body movements. Then rats were placed in their home cage for 2 h.

### Rectal or colorectal distension and colonic hypersensitivity measurement. 

Mice colonic sensitivity induced by NMS was assessed by quantifying visceromotor response (VMR) with abdominal electromyogram recordings in response to colorectal distension (CRD), as previously described^18^.

Rats colonic sensitivity induced by PRS was assessed by quantifying electrical response through an electroencephalograph Reega Mini-hui (ALVAR, France) and expressed as number of abdominal cramps for a five min period as previously described^19^. Briefly, rats were accustomed to be in polypropylene tunnels (diameter 7 cm, length 20 cm) several days before CRD in order to minimize recording artifacts. CRD was performed with an arterial embolectomy catheter (Fogarty; Edwards Laboratoire, Inc., Santa Ana, CA, USA) introduced into the rectum (1 cm from the anus) and fixed at the base of the tail. Distension of the colon was performed by connecting the catheter to a syringe and consecutive injections of different volumes (0.4, 0.8, 1.2 ml) with an interval of 5 minutes. Each animal was recorded two days before the stress (basal measure) and just after the PRS (stress measure).

### Fecal pellets collection and DNA extraction

Fecal pellets were collected directly from mice at week 3 and week 12 and stored at −80 °C prior to DNA extraction. Bacterial DNA was extracted from fecal bacteria following the protocol of NucleoSpin® Soil kit (Macherey-Nagel, Düren, Germany). DNA concentrations and purity were then assessed using Take3 micro-volume plate and Epoch Microplate Spectrophotometer (BioTek, Winooski, VT, USA).

### Sequencing

The 16 S rRNA gene V4 variable region PCR primers 515/806 with barcode on the forward primer were used in a 30 cycle PCR (5 cycle used on PCR products) using the HotStarTaq Plus Master Mix Kit (Qiagen, Germantown, MD, USA). Next generation sequencing (NGS) was performed at Molecular Research DNA (MR DNA - Shallowater, TX, USA) on a MiSeq following the manufacturer’s guidelines. Sequence data were processed using MR DNA analysis pipeline (MR DNA, Shallowater, TX, USA).

### Tissue preparation for histological analysis

Flushed colons were opened longitudinally, cut into 2 cm sections and rolled according to swiss-roll procedure^20^. The samples were fixed in 4% paraformaldehyde (24 h, 4 °C), dehydrated, and embedded in paraffin according to standard histological protocols. Four-micrometer sections of distal colon were mounted on SuperFrost® Plus slides. To assess colonic damage, the sections were stained with hematoxylin/eosin and examined blindly according to the Ameho criteria^21^.

### Cytokine analysis

Before sacrifice, blood samples were obtained from the retro-orbital venous plexus, centrifuged and stored at −80 °C. Cytokine levels were determined by Flow Cytometry using Cytometric Bead Array analysis (Mouse Inflammation Kit,BD, NJ, USA).

### Immuno-modulatory properties using HT-29 cells

Anti-inflammatory assays were done following the procedure described by Kechaou *et al*.^22^. Briefly, 50000 HT-29 cells per well were seeded in 24-wells culture plates (Nunc) in DMEM media (Lonza) supplemented with 10% FBS (Lonza) and 1% glutamine (Sigma-Aldrich). Experiments were initiated on day 7 after seeding, when cells were at confluence (1.83 × 10^6^ cells/well). Twenty-four hours before bacterial co-culture (day 6), the culture medium was changed for a medium with 5% heat-inactivated FBS and 1% glutamine. On the day of co-culture, 10% of bacterial supernatant were added in DMEM in a total volume of 500 μl. Cells were stimulated simultaneously with human TNF-α (5 ng/ml; Peprotech, NJ) for 6 h at 37 °C in 10% CO_2_. All samples were analyzed in triplicate. After co-incubation, cell supernatants were collected and stocked at −80 °C until further analysis of interleukin-8 (IL-8) concentrations by ELISA (Biolegend, San Diego, CA). Total protein was determined by Bradford Reagent test (Sigma-Aldrich). Experiments have been done at least in triplicate. Results are expressed as IL-8/ protein (pg/mg) and have been normalized using as reference value the IL-8 produced after the co-incubation with PBS as a negative control.

### *In vivo* intestinal permeability

*In vivo* intestinal permeability was assessed using fluorescein dextran (FITC-dextran 3000–5000 Da, Sigma-Aldrich) as previously described^23^. Briefly, before and after *F. prausnitzii* treatment period, mice were orally gavaged with 0.6 mg/g body weight of FITC-dextran and blood samples were obtained from the retro-orbital venous plexus 3.5 h after this administration. Plasma FITC levels were determined by fluorometry at 488 nm using a microplate reader (Tecan, Lyon, France).

### Immunohistochemistry

Colon were embedded in OCT medium and stored at −80 °C. Ten micrometers of frozen colon were cut in a cryostat. Colonic mucosa were fixed in 1% paraformaldehyde (PFA) for 20 minutes, washed in PBS, and permeabilization was performed using 0.5% Triton X-100 in PBS for 20 minutes. Unspecific sites were blocked using PBS 5% goat serum and 2% bovine serum albumin (BSA) for 1 hour. Rabbit polyclonal occludin antibody (#71-1500, Invitrogen) or rabbit polyclonal to Claudin-2 – Aminoterminal end antibody (#ab76032, AbCam)^24^ was diluted in blocking buffer (1/500 and 1/300 respectively) and incubated overnight at 4 °C. After PBS washes, tissues were incubated for 90 minutes with an Alexa Fluor 546 goat anti-rabbit (Invitrogen) secondary antibody diluted in PBS 5% goat serum supplemented with Alexa Fluor 488 phalloidin (Invitrogen). Slides were mounted using Fluorescent Mounting Medium (Dako). Tissues were visualized using a microscope Nikon Eclipse Ni-E and the NIS-Elements analysis software (Nikon Instruments Inc., Melville, NY). The mean of fluorescence intensity (MFI) and the area of colonic tissue (area) of each imagen was measured using Image J Software. A total of four images were considered for each mice.

### Statistical Analysis

Statistical analysis was completed using GraphPad software (GraphPad Sofware, La Jolla, CA, USA). Results are presented with means ± SEM except for Illumina experiments, median ± SEM. For mice experiments, sensitized (mother-separated) mice displaying VMR values lower than mean minus two standard errors for all distension volumes were considered as non-sensitized and excluded from the analysis (Grubb’s Test). Differences in VMR to gradual CRD were analyzed using a 2-way ANOVA (Treatment, Volume) followed by Bonferroni *post-hoc* test for multiple comparisons. Rate comparisons were performed using Fisher’s exact test. Most comparisons were performed by One-Way analysis of variance followed by the Student-Newman-Keuls multiple comparison post hoc analysis. For data sets that were non-Gaussian or based on a score or on a percentage, data were compared using the non-parametric test Kruskal-wallis followed by a Dunn’s Multiple Comparison *test*. The values of CRD were analyzed using the Mann Whitney U *test* for unpaired data and the Wilcoxon test for paired data. A *p* value of less than 0.05 was considered significant.

## Results

### Microbiota abundance of *F. prausnitzii* impacts on colonic hypersensitivity and permeability in a non-inflammatory NMS-induced murine model

Next generation sequencing (NGS) of the 16 S genes were performed by Illumina on fecal samples of NMS mice compared to control non handled (NH) mice, just after weaning (Week 3) and at the CRD period (Week 12), to analyze the composition of the microbiota. At the phylum level, such NGS analysis indicated that the microbiota of NMS mice and control NH mice were quite similar (data not shown). Principal component (PCoA) plots of unweighted UniFrac analysis also demonstrated any significant differences in the species level global composition of NMS mice and control NH mice (data not shown). Together, these results suggest that NMS paradigm did not result in marked alterations of the gut microbiota.

**Figure 2:**
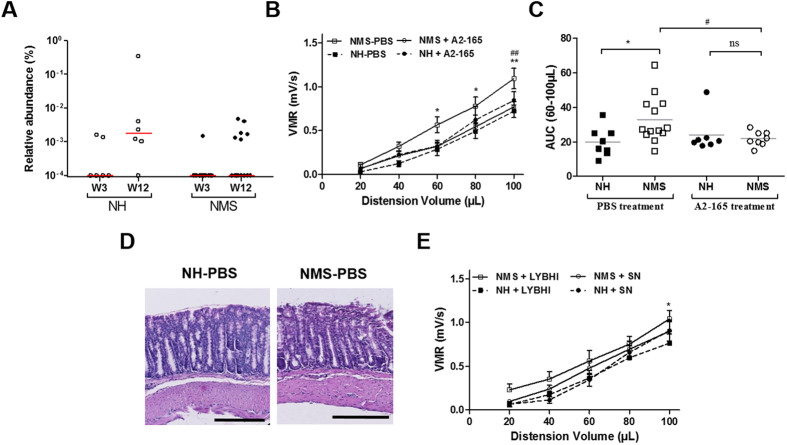
Microbiota abundance of *F. prausnitzii* impacts on colonic hypersensitivity in a non-inflammatory NMS-induced murine model. (**A**) Median of relative abundance of *F. prausnitzii* in percentage of operational taxonomic unit (OTU) of *F. prausnitzii*-like 16 S sequence detected by Next generation sequencing (NGS) Illumina on fecal samples of NMS mice compared to NH mice, just after weaning (Week 3 (W3), n = 14 and n = 6 respectively) and at the CRD period (Week 12 (W12), n = 14 and n = 6 respectively). (**B**) Visceromotor response (VMR) to colorectal distension (CRD) in control (PBS) NH and NMS sensitized mice (n = 8 and n = 13 respectively) and *F. prausnitzii* treated mice (A2-165) (n = 7 and n = 8 respectively). (**C**) The area under the curve (AUC) were calculated between 60 μL and 100 μL for the different mice groups. (**D**) Representative colonic tissue sections from NH and NMS sensitized mice control (PBS). (**E**) VMR to CRD in NH and NMS sensitized mice control (LYBHI) (n = 8 and n = 7 respectively) and *F. prausnitzii* supernatant (SN) treated (n = 7 and n = 7 respectively). Concerning statistical analysis: * represents statistical differences between groups with the same treatment but different stress (NH versus NMS) and ^#^ represents statistical differences between groups with the same stress but different treatments (PBS versus A2-165 or SN versus LYBHI); ns: no significant difference; * or ^#^*P* < 0.05; ** or ^##^*P* < 0.01.

To determine if microbiota relative abundance of *F. prausnitzii*-like specie could be impacted by a NMS stress, we further mined our NGS data to examine if operational taxonomic unit (OTU) of *F. prausnitzii*-like 16 S sequence (99% match) could be observed. Such OTU was detected in a few amounts of mice just after weaning (33% and 7% of NH and NMS mice, respectively). In adults, its relative abundance in fecal microbial population was very low (0.003% in NH mice and 0.001% in NMS mice) showing a trend of 2.6-fold decrease (Fig. 2A). In addition, the rate of adult mice harboring *F. prausnitzii*-like OTU was significantly decreased in NMS mice (42%) compared to NH mice (83%) (Fisher’s exact test P < 0.001), suggesting a negative impact of the NMS stress on *F. prausnitzii* population of fecal microbiota.

Neonatal Maternal Separation induced a significant visceral hypersensitivity up to 8 weeks after separation paradigm. When compared to non-handled control mice (NH-PBS), NMS control mice (NMS-PBS) exhibited a significant increase in visceromotor response (VMR) to CRD, for the highest distension volumes, reflecting CHS (Fig. 2B,C). In this model, colonic histological examination did not show inflammatory changes (Fig. 2D). Moreover, the other evaluated parameters such as body weight, or spleen weight, colon weight, colon length and Ameho score did not show sign of inflammation (Table S1). Finally, cytokines quantification (Figure S1A,B) confirmed the absence of systemic inflammation in NMS mice.

*F. prausnitzii* A2-165 and its SN effects were tested as curative treatment, by a daily oral gavage during 10 days, on the CHS in the NMS-induced mouse model. *F. prausnitzii* treatment significantly (P < 0.05) reduced VMR to CRD in comparison to NMS-PBS mice for the highest distension volumes, reaching a level similar to NH-PBS mice (Fig. 2B,C). The culture SN of *F. prausnitzii*, presenting anti-inflammatory properties (Figure S1C), did not reverse CHS induced by NMS paradigm (Fig. 2E).

**Figure 3:**
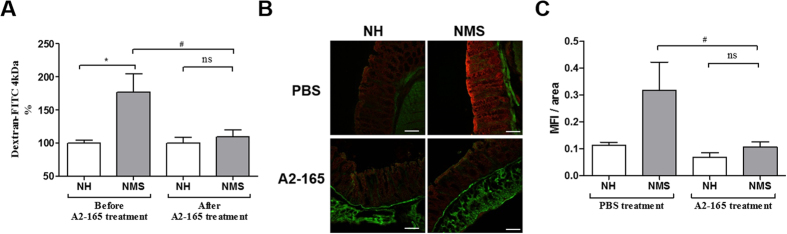
*F. prausnitzii* impacts colonic permeability in NMS-induced murine model with barrier dysfunction. (**A**) *In vivo* measurements of gut permeability, animals were orally inoculated with FITC-dextran before and after PBS or *F. prausnitzii* treatment (*n* = 16 mice per group). (**B**) Representative sections of the distal colon; Claudin-2 (red) and Phalloidin (green) immunostaining (bare scale represents 100 μm). (**C**) Ratio of the mean of fluorescence intensity of Claudin-2 (MFI) and the area of colonic tissue (area), quantified in each groups: control (PBS) NH and NMS sensitized mice (n = 4 and n = 5 respectively) and *F. prausnitzii* treated mice (A2-165) (n = 3 and n = 4 respectively). Concerning statistical analysis: * represents statistical differences between groups with the same treatment but different stress (NH versus NMS) and ^#^ represents statistical differences between groups with the same stress but different treatments (PBS versus A2-165); ns: no significant difference; * or ^#^*P* < 0.05.

Intestinal permeability was assessed using the paracellular tracer FITC-dextran. NMS induced a significant increase of intestinal permeability in comparison to NH-PBS mice (Fig. 3A). After 10 days of *F. prausnitzii* oral treatment, this gut barrier defect was significantly reversed (Fig. 3A). Pore-forming claudin-2 expression, but not occludin, was increased in colon of NMS-PBS mice compared to NH-PBS mice (Fig. 3B and Figure S1D). *F. prausnitzii* A2-165 treatment restored significantly the expression of the pore-forming claudin-2 (Fig. 3B,C).

### *F. prausnitzii* prevents visceral hypersensitivity in a model of Partial Restraint Stress

**Figure 4:**
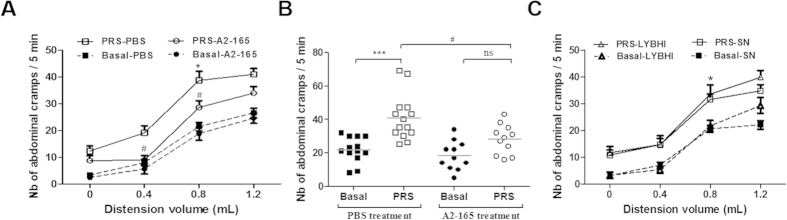
Effect of *F. prausnitzii* and its supernatant on visceral hypersensitivity in PRS model. Colorectal distension measures were taken 2 days before PRS (basal) and after PRS. (**A**) Effect of *F. prausnitzii* A2-165 strain (A2-165) (n* = *11 rats per group) compared to control group (PBS) (at least n* = *13 rats per group). (**B**) Detail of the results at 0.8 mL. (**C**) Effect of *F. prausnitzii* A2-165 supernatant (SN) compared to culture medium (LYBHI). Results are expressed as number of abdominal cramps for each five minutes period. Concerning statistical analysis: * represents statistical differences between groups with the same treatment but different stress (Basal versus PRS) and ^#^ represents statistical differences between groups with the same stress but different treatments (PBS versus A2-165); ns: no significant difference; * or ^#^*P* < 0.05; *** or ^###^*P* < 0.001.

To determine if *F. prausnitzii* and/or its SN are able to prevent acute stress generated symptoms on visceral sensitivity, both were tested on a model of Partial Restraint Stress. PRS increased the number of abdominal cramps in response to CRD in a volume-dependent manner (Fig. 4A,B). In stressed rats treated with PBS, the first volume of distension that significantly increased the number of abdominal contractions compared to non-stressed animals was 0.8 mL (*p <* 0.05) (Fig. 4A,C). *F. prausnitzii* treatment prevented this stress-induced visceral hypersensitivity (*p* < 0.05) (Fig. 4A,B). In basal conditions, no difference was observed in the VMR to CRD between the different treatments (Fig. 4A,B). In contrast to *F. prausnitzii*, the SN did not show a protective effect (Fig. 4C).

## Discussion

*Faecalibacterium prausnitzii* is a widely distributed bacteria in the more anaerobic part of GI tract of mammals as rodents with different type of phylotypes^25^. In the present study, NGS of the mouse fecal microbiota revealed that *F. prausnitzii* OTU was detected in few amount of 3 week-old control mice (33%), since it was more frequently observed in 12 week-old control mice (83%), to represent 0.003% of fecal microbiota. Such result was consistent with kinetic of implantation of this specie observed in GI tract of humans^26^. Interestingly, the rate of adult mice harboring *F. prausnitzii* OTU was significantly decreased in NMS mice (42%) in comparison to control NH mice (83%). The stress induced by the NMS paradigm is known to be an early traumatic experiences having long-term consequences on gastrointestinal functions as immune status, sensitivity, motility and permeability, and representing a pathophysiological model for IBS^27^. Thus, such long-term dysfunctions could also impact on *F. prausnitzii* relative abundance in the gut microbiota.

IBS disorder may be classified according to the predominant bowel habit into IBS-D (diarrhea), IBS-C (constipation) IBS-A (alternating)^28^. In some patients, these symptoms appear after transient exposure to a GI pathogen. This entity is called post-infective (PI)-IBS, and is characterized by the clearance of the triggering microorganisms but persistence of the gut dysfunction or of low-grade inflammation^29^. The high heterogeneity of this syndrome makes very difficult to make conclusions. An example of the heterogeneity of IBS is the diversity of the abundance of *F. prausnitzii*: its abundance is low in cases of IBS-A^8^, unaffected in cases of IBS-D and may or may not be lower than in IBS-C patients than healthy controls^30^,^31^,^32^,^33^. An instance, negative correlation between beneficial species of the gut and IBS symptoms have been demonstrated with Rajilić-Stojanović *et al*. reporting a reduction in *Faecalibacterium spp*., in faecal samples, being associated with an increase in IBS symptoms^8^. However, a recent study by Pozuelo *et al*. did not reproduce the same tendencies although a reduction of butyrate producing bacteria was found in IBS-D and IBS-A patients^34^. These observations confirmed the importance of personalized therapy with a patients’ stratification based on *F. prausnitzii* relative abundance in the gut microbiota. Such analysis could be performed on fecal sample, a non-invasive taking, that represents colon microbiota: *F. prausnitzii* niche’s^11^.

To evaluate the effect of *F. prausnitzii* and its culture SN on visceral sensitivity in response to CRD, the VMR was measured in two different models of stress (a chronic stress induced by NMS and an acute stress episode of PRS), and in two different rodent species (mice and rats). Changes in the VMR, a pseudo-affective reflex response widely used as index of visceral pain in rodents, depends on changes on the pressure inside the colonic lumen resulting from changes of the gut wall compliance. In agreement with this, previous investigations were conducted with the barostat technique (Distender Series II Barostat, G&J Electronics) in order to evaluate the gut wall compliance changes in various stress models in rats, including the PRS. Results have shown that the PRS applied in female Wistar rats (same protocol as this used herein) increased stress-induced visceral sensitivity in absence of any effect on gut compliance^14^,^35^,^36^,^37^. Based on these results, we^38^ and others^39^ have published later by monitoring the visceral hypersensitivity induced by PRS with the method used in this study. In the same way, in the mice NMS stress model, modified response to CRD was validated using a barostat system for pressure-controlled, graded CRD. In agreement with previous findings, both PRS or NMS stress induce visceral hypersensitivity in rodents^13^,^14^. Treatment of stressed animals with *F. prausnitzii*, but not with SN, has curative anti-nociceptive properties underlying that this beneficial effect is provided by the complete bacteria and suggesting multi-component action mechanisms. Taking into account i) that *F. prausnitzii* did not affect visceral sensitivity in basal conditions in two different rodent models and ii) that the PRS or NMS stress had no effect on gut compliance, the anti-nociceptive effects of *F. prausnitzii* treatment did not result from changes in colonic compliance. *F. prausnitzii* has been shown to be able to colonize lower part of the GI tract and to impact colonic epithelial homeostasis^40^. In addition, *F. prausnitzii* can modulate tight junctions in animal models with low grade or acute inflammation^41^,^42^. The present study confirm that a chronic stress, as it was already shown for an acute stress^14^, result in barrier dysfunction in rodents. Interestingly, both anti-inflammatory and anti-nociceptive effects of *F. prausnitzii* seem to be associated with a preservation of tight junction integrity. Hypersensitivity to rectal or colonic distension characterizes most patients with IBS and increased gut permeability has been described in post-dysenteric IBS patients. A cause-and-effect relationship between intestinal hyper-permeability and visceral hypersensitivity in the PRS rat model was established^35^. In fact, the increase of colonic permeability results from epithelial cell cytoskeleton contraction through myosin light chain (MLC) phosphorylation via MLC kinase activation and this increase is responsible for stress-induced rectal hypersensitivity. Moreover, a prevention of this hyper-permeability by decrease of the phosphorylation was shown for other bacteria, as the probiotics strain *Lactobacillus farciminis*^14^. Clinical data supports these results; besides a decrease of *F. prausnitzii* in IBS patients’ microbiota^9^, and an increase of intestinal permeability may lead to more severe IBS symptoms and hypersensitivity to somatic and visceral stimuli^43^.

Using non-inflammatory models, this study demonstrates that beyond anti-inflammatory properties^10^,^11^,^12^, *F. prausnitzii* is also able to reduce visceral hypersensitivity induced by stress possibly through intestinal epithelial barrier enhancement. This commensal bacterium which presents a diminished prevalence in many patients suffering from different intestinal disorders, could thus be considered as a keystone specie of intestinal health^7^. These data support the increasing interest in developing microbiota-modulating therapies for patients suffering from intestinal disorder and the use of commensal bacterium as a next-generation probiotics^44^.

## Additional Information

**How to cite this article**: Miquel, S. *et al*. Anti-nociceptive effect of *Faecalibacterium prausnitzii* in non-inflammatory IBS-like models. *Sci. Rep*. **6**, 19399; doi: 10.1038/srep19399 (2016).

## Supplementary Material

Supplementary Information
