# Supplementary material for: Anti-nociceptive effect of Faecalibacterium prausnitzii in non-inflammatory IBS-like models
Source: Sci Rep. 2016 Jan 18;6:19399. doi: 10.1038/srep19399 (PMC4726104; doi:10.1038/srep19399)
Supplement: Supplementary Information [file srep19399-s1.pdf]

Anti-nociceptive effect of *Faecalibacterium prausnitzii* in non-inflammatory IBS-like models

S. Miquel[1,2,3¥,4¥#]*, R. Martín[1,2#], A. Lashermes[5,6], M. Gillet[7], M. Meleine[5,6], A. Gelot[5,6], A. Eschalier[5,6], D. Ardid[5,6], L. G. Bermúdez-Humarán[1,2], H. Sokol[1,2,8,9,10,11], M. Thomas[1,2], V. Theodorou[7], P. Langella[1,2$], F. A. Carvalho[5,6, $,*]

SUPPLEMENTARY INFORMATIONS

Supplemental Table 1: Intestinal health parameters. Non-handled control mice (NH-PBS) and (NH-LYBHI), Neonatal Maternal Separation control mice (NMS-PBS) and (NMS-LYBHI), Non-handled *F. prausnitzii* (NH- A2-165) or supernatant (NH- SN) treated mice, NMS *F. prausnitzii* (NMS- A2-165) or supernatant (NMS- SN) treated mice AU: arbitrary units.

	Body weight (g)	Spleen weight (mg)	Colon weight (mg)	Colon lenght (cm)	Ameho score (AU)
NH-PBS	22.8+/-0.3	64.7+/-3.5	221.6+/-6.2	8.3+/-0.1	0
MS-PBS	22.4+/-0.4	76.7+/-4.7	215.4+/-8.5	8.1+/-0.2	0
NH-A2-165	23.2+/-0.5	70.0+/-3.4	223.1+/-6.6	8.3+/-0.3	0
MS-A2-165	22.2+/-0.4	69.0+/-3.1	216.4+/-6.0	7.8+/-0.2	0
NH-LYBHI	22.3+/-0.6	68.2+/-3.4	224.5+/-8.8	8.1+/-0.2	0
MS-LYBHI	22.1+/-0.7	74.1+/-3.6	213.8+/-7.1	8.1+/-0.2	0
NH-SN	22.0+/-0.5	71.6+/-3.8	214.7+/-7.6	7.7+/-0.2	0
MS-SN	21.9+/-0.4	79.9+/-6.0	212.1+/-4.4	7.9+/-0.3	0

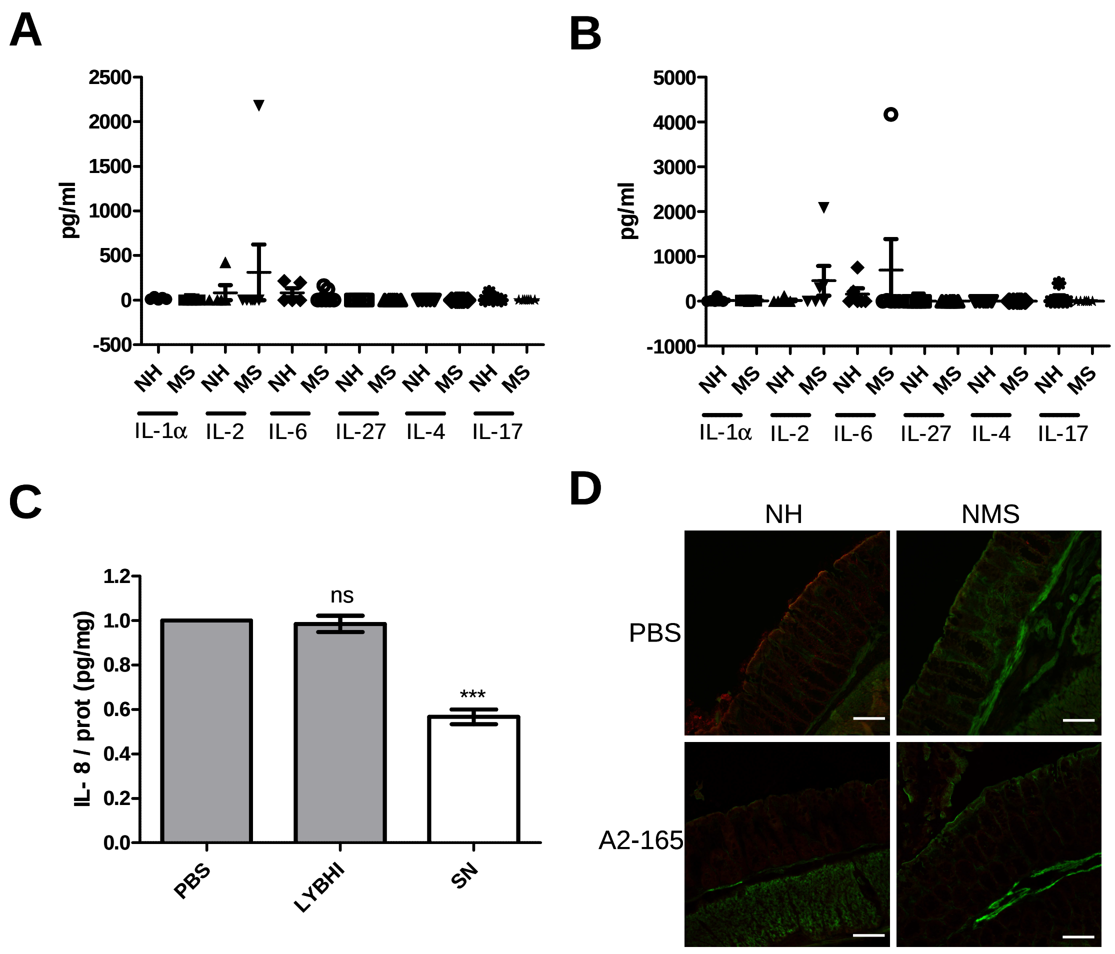

Figure S1: Quantification of plasma cytokine levels in NH mice compared to NMS mice in **(A)** control group (PBS) and **(B)** *F. prausnitzii* treated. **(C)** Immuno-modulation capacities of *F. prausnitzii* A2-165 supernatant (SN) *in vitro.* IL-8 production in HT-29 TNF-α stimulated cells. Experiments have been done at least in triplicate. Results are expressed as IL-8/ quantity of protein (pg/mg) and have been normalized using as reference value the IL-8 produced after the co-incubation with PBS as a negative control. **(D)** Sections of the distal colon were stained for Occludin (red) and Phalloidin (green) expression. Original magnification X200 (bare scale represents 100µm). Representative images from each group. *** P<0.001.
